# Subcutaneous Tocilizumab May Be Effective in Refractory Fibromyalgia Patients

**DOI:** 10.3390/biomedicines11071774

**Published:** 2023-06-21

**Authors:** Kuo-Tung Tang, Tsai-Ling Liao, Yi-Hsing Chen, Der-Yuan Chen, Kou-Lung Lai

**Affiliations:** 1 Division of Allergy, Immunology, and Rheumatology, Taichung Veterans General Hospital, Taichung 407, Taiwan; dirac1982@vghtc.gov.tw (K.-T.T.); ysanne@vghtc.gov.tw (Y.-H.C.); 2 Faculty of Medicine, National Yang Ming Chiao Tung University, Taipei 112, Taiwan; 3 Ph.D. Program in Translational Medicine, Rong Hsing Research Center for Translational Medicine, National Chung Hsing University, Taichung 402, Taiwan; tlliao1972@gmail.com (T.-L.L.); dychen1957@gmail.com (D.-Y.C.); 4 Department of Medical Research, Taichung Veterans General Hospital, Taichung 407, Taiwan; 5 Department of Post-Baccalaureate Medicine, College of Medicine, National Chung Hsing University, Taichung 402, Taiwan; 6 Rheumatology and Immunology Center, China Medical University Hospital, Taichung 404, Taiwan; 7 College of Medicine, China Medical University, Taichung 404, Taiwan; 8 Institute of Medicine, Chung Shan Medical University, Taichung 402, Taiwan

**Keywords:** fibromyalgia, neutrophils, pain, tocilizumab

## Abstract

Introduction: Fibromyalgia (FM) is a chronic disorder characterized by widespread pain with an enormous symptom burden. Its treatment efficacy is limited. Its pathogenesis involves immune dysregulation, which includes interleukin-6 (IL-6) production. Methods: We herein reported a case series of FM patients receiving subcutaneous tocilizumab at our institution. FM symptoms were evaluated by the revised Fibromyalgia Impact Questionnaire (FIQR), which included pain level, and the fibromyalgianess scale based on the 2016 criteria of the American College of Rheumatology (ACR). FM symptoms were compared using the Wilcoxon signed-rank test. Neutrophils from primary FM patients and matched healthy controls were also isolated for transcriptome analysis. Results: We presented a total of two primary and four secondary FM patients who had received subcutaneous tocilizumab for a minimum of 12 weeks. All patients had severe symptoms despite standard treatments. Patients’ FIQR and fibromyalgianess both dropped at 4 and 12 weeks. Four (67%) of them reached a pain reduction of ≥30% at 4 weeks, and three (50%) reached a pain reduction of ≥30% at 12 weeks. Possible differentially expressed genes were identified in primary FM patients when compared with controls and after tocilizumab treatment. Conclusions: FM patients likely benefited from subcutaneous tocilizumab therapy. A randomized controlled trial is needed to verify its efficacy.

## 1. Introduction

Fibromyalgia (FM) is a debilitating disorder characterized by widespread pain. The pathogenesis of FM remains elusive. The dysfunctional processing of pain sensation in the brain contributes to its pathogenesis. Prior studies on FM patients revealed their higher blood levels of pro-inflammatory cytokines, including interleukin (IL)-6 [1]. In particular, IL-6 induces hyperalgesia, fatigue, and depression in mice [2,3]. A recent study on mice showed that IgG extracted from FM patients generated animals’ nociceptive hypersensitivity [4]. Furthermore, patients with rheumatic diseases have a higher prevalence of concomitant FM. It is likely that the immune system plays a crucial role in the FM symptoms of these patients.

To date, the benefits of FM treatments are modest, often with questionable clinical relevance [5]. The participation of the immune system in the generation of pain has been implicated. Among immune cells, migrating neutrophils, partly through their elastase activity, contribute to the development of pain, as shown in mice [6,7]. Of note, IL-6 inhibition alleviates pain independent of arthritis amelioration in patients with rheumatoid arthritis (RA) [8]. Furthermore, IL-6 inhibition could suppress the activation of neutrophils in humans [9]. Tocilizumab, a humanized monoclonal antibody against IL-6 receptor, has been shown to be effective against a variety of inflammatory diseases, including RA, juvenile idiopathic arthritis, adult-onset Still’s disease, giant cell arteritis and Takayasu arteritis [10]. Nevertheless, clinical studies on IL-6 inhibition in FM patients are basically unavailable.

In our institution, we have so far treated six FM patients using tocilizumab. Here, we presented their relevant features.

## 2. Materials and Methods

### 2.1. Study Participants

From December 2020 through September 2022, a total of 2 primary and 4 concomitant FM patients, diagnosed based on the 1990 American College of Rheumatology (ACR) classification criteria, received subcutaneous tocilizumab at our hospital. In addition to these 2 primary FM patients, another 3 primary FM patients and 5 healthy controls with matched age and sex were enrolled for the comparison of neutrophil transcriptomes. This study complied with the Declaration of Helsinki, and was approved by the Institutional Review Board of Taichung Veterans General Hospital (approval No. CE21042A). All study subjects were Han Chinese, and written informed consent was obtained.

### 2.2. Laboratory Examinations and 40-Joint Sonography

Erythrocyte sedimentation rate (ESR) was determined using the SRS 20/II autoanalyzer (Vacuette, Greiner Bio-One GmbH, Kremsmünster, Austria) with the Westergren method. The normal ranges were 0–10 mm/h for males and 0–20 mm/h for females. High-sensitivity C reactive protein (hsCRP) was evaluated using immunoturbidimetry (Wako Pure Chemical Industries, Ltd., Osaka, Japan). Its normal range was below 0.3 mg/dL. Each FM patient underwent 40-joint sonography at baseline to detect the presence of occult synovitis based on a standardized protocol. The joints we studied included the following: bilateral shoulders, elbows, wrists, 5 metacarpophalangeal joints, thumb interphalangeal joints, 4 proximal interphalangeal joints, knees, ankles, and 5 metatarsophalangeal joints. Sonography was performed by an experienced technician using the Philips iU22 (Philips Ultrasound, Inc., Bothell, WA, USA) equipped with a 12 MHz linear transducer with power Doppler function. The pulse repetition rate was 500 Hz. The grading of synovitis was interpreted by a rheumatologist (KL Lai), who had 15 years of experience in musculoskeletal ultrasound, based on the criteria of Outcome Measures in Rheumatology (OMERACT) [11]. The maximum grade of either effusion or synovial hypertrophy was ascribed to the grading of grey-scale imaging. Grade 0/1 on the grey-scale imaging and grade 0 (no signal) on the power Doppler imaging were considered normal [12]. Patients received 40-joint sonography one more time 12 weeks after tocilizumab therapy.

### 2.3. Clinical Parameters

To evaluate disease severity of FM patients, we applied the Chinese version of revised Fibromyalgia Impact Questionnaire (FIQR) (Cronbach’s α: 0.95, validated in 103 Taiwanese FM patients; Whei-Mei Shih, personal communication) [13]. The levels of pain, energy, and sleep quality were evaluated by the answers to the FIQR questions, each based on an 11-point numeric rating scale from 0 to 10. We asked each patient to complete the questionnaire, first at baseline and then at 4 and 12 weeks after tocilizumab therapy.

Each patient also completed another questionnaire based on the 2016 revision to the 2010/2011 ACR fibromyalgia criteria. The questionnaire has two main components: (a) the widespread pain index (WPI) and (b) the symptom severity (SS) score. The WPI and SS scores were summed to derive a fibromyalgianess scale, which indicated the severity of FM. The questionnaire was completed at baseline and 4 and 12 weeks after tocilizumab therapy. 

### 2.4. Neutrophils Isolation

Neutrophils were isolated from venous blood using Polymorphprep (Axis-Shield, USA), according to the manufacturer’s instructions. After centrifugation at 500× *g* for 30 min at 25 °C with low brake, neutrophils sunk to the middle polymorphonuclear leukocyte (PMN) layer of the solution. The PMN layer was transferred to a clean tube. Then, ACK lysis buffer (154 mM NH_4_Cl, 10 mM KHCO_3_, and 1 mM EDTA) was added and mixed gently. After 5 min, the mixture was centrifuged at 500× *g* for 5 min at 25 °C. Pellets were suspended in 10 mL Hank’s balanced salt solution (HBSS, Sigma-Aldrich, St. Louis, MO, USA) and dispersed gently. After centrifugation at 500× *g* for 5 min at 25 °C, neutrophil pellets were obtained and suspended in 1 mL HBSS.

### 2.5. RNA Extraction from Neutrophils for Transcriptome Analysis

Total RNAs were extracted from isolated neutrophils using the TRIzol Reagent (Thermo Fisher Scientific, Waltham, MA, USA) and purified using a GENEzol TriRNA Pure kit (Geneaid, Taiwan R.O.C.), according to the manufacturer’s instructions. Purified RNAs were quantified at OD260 and 280 nm using a NanoDrop spectrophotometer (Thermo Fisher Scientific). RNA sequencing data were deposited in Gene Expression Omnibus (GEO) under GEO accession number GSE229750.

### 2.6. Library Preparation and Sequencing

The purified RNA was used for the preparation of the sequencing library using the TruSeq Stranded mRNA Library Prep Kit (Illumina, San Diego, CA, USA) according to the manufacturer’s instructions. In brief, mRNA was purified from total RNA (1 μg) using oligo(dT)-coupled magnetic beads and fragmented into small pieces at an elevated temperature. The first-strand cDNA was synthesized using reverse transcriptase and random primers. After generation of double-stranded cDNA and adenylation on 3′ ends of DNA fragments, adaptors were ligated and purified using the AMPure XP system (Beckman Coulter, Beverly, MA, USA). The quality of the libraries was assessed with the Agilent Bioanalyzer 2100 system and a real-time PCR system. The qualified libraries were then sequenced on an Illumina NovaSeq 6000 platform using 150 bp paired-end reads (Genomics, BioSci & Tech Co., New Taipei City, Taiwan).

### 2.7. Bioinformatics Analysis

Bases with low quality and sequences from adapters in raw data were removed using program Fastp (version 0.20.0). Filtered reads were aligned to the reference genomes using the HISAT2 software (version 2.1.0). The software FeatureCounts (v2.0.1) in Subread package was applied to quantify the gene abundance. Differentially expressed genes (DEGs) were identified using DESeq2 (version 1.28.0) or EdgeR (version 3.36.0).

### 2.8. Statistical Analyses

Numerical data were presented as median ± interquartile range (IQR). Categorical data were presented in percentages. Comparisons of numerical variables across time points were made using the Wilcoxon signed-rank test. All statistical analyses were performed on the software Stata, version 14.0 (StataCorp, College Station, TX, USA).

## 3. Results

### 3.1. Baseline Characteristics

FM patients’ baseline characteristics are presented in Table 1. Most of them (83%) were female. The four concomitant FM patients each had rheumatoid arthritis, hypereosinophilic syndrome, ankylosing spondylitis, or Sjogren’s syndrome. Their median age was 51 years (IQR: 45 and 52 years), and their tender points were 18 (IQR: 16 and 18). Four (67%) of them had depression. Their baseline pain level was 10 (IQR: 8.5 and 10), and disease duration was 3.5 (IQR: 3 and 5.5) years. At baseline, their fibromyalgianess scale was 27.5 (IQR: 26 and 30) and FIQR score was 80 (IQR: 74 and 88). In terms of medications, four received pregabalin 450 mg/day, and two received duloxetine 60 mg/day. All of the patients had refractory symptoms despite medications. At baseline, only one patient had bilateral elbows with mild synovitis based on results from the 40-joint sonography.

### 3.2. The Therapeutic Effects of Tocilizumab in FM Patients

**Figure 1:**
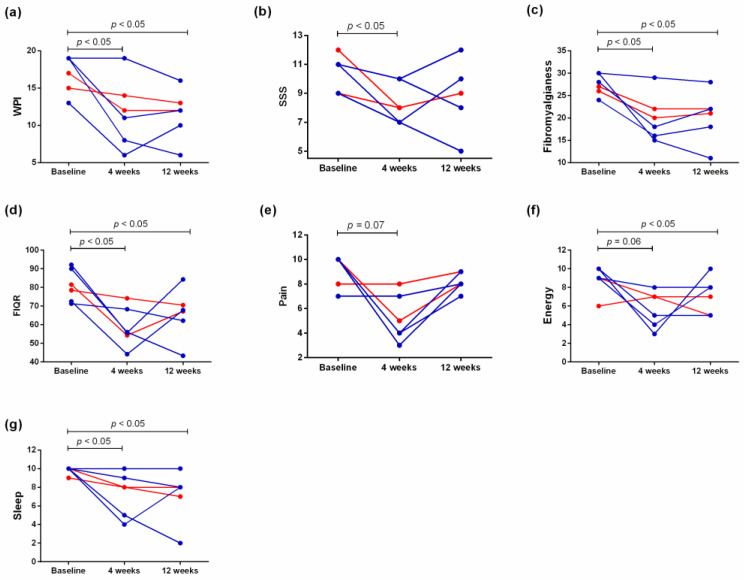
Comparison of (**a**) widespread pain index (WPI), (**b**) symptom severity score (SSS), (**c**) fibromyalgianess score, (**d**) the revised Fibromyalgia Impact Questionnaire (FIQR) score, (**e**) level of pain, (**f**) level of energy, and (**g**) sleep quality between baseline, 4, and 12 weeks after subcutaneous tocilizumab therapy in fibromyalgia patients. The red lines denote two primary fibromyalgia patients, and the blue lines denote four concomitant fibromyalgia patients.

Of the six FM patients, four received subcutaneous tocilizumab 162 mg every 2 weeks, and two received tocilizumab 162 mg every 4 weeks (Table 1). As shown in Table 2 and Figure 1, the patients’ FIQR and fibromyalgianess dropped at both 4 and 12 weeks. Their pain levels showed a decreasing trend at 4 weeks. Four (67%) patients achieved ≥30% pain reduction at 4 weeks, and three (50%) achieved ≥30% pain reduction at 12 weeks. The patients’ energy and sleep quality both improved at 12 weeks. In terms of joint inflammation evaluated by the 40-joint sonography, we found no significant change (Table S1). 

In terms of adverse events, there was no infection among all patients during the 12-week treatment period. There was an elevated level of low-density lipoprotein cholesterol in one patient at 12 weeks after tocilizumab therapy.

### 3.3. Differentially Expressed Genes in Neutrophils of Primary FM Patients

We compared the transcriptome of neutrophils in five female primary FM patients (including patients 1 and 2; mean age 51 ± 1 years) and five matched healthy controls. Two genes upregulated in primary FM patients were *C3AR1* (4-fold) and *PI3* (2-fold) when compared with healthy controls (Table S2). 

### 3.4. Effects of Tocilizumab on Gene Expression Profile of Neutrophils in Primary FM Patients

In the two primary FM patients who underwent subcutaneous tocilizumab therapy, we found, at 12 weeks after treatment, 23 overlapping differentially expressed genes (fold change >2) in their neutrophils (Figure S1 and Table S3). In particular, *SPP1* was down-regulated in their neutrophils after tocilizumab therapy.

## 4. Discussion

FM is a distressful disorder, partly due to the modest therapeutic efficacy of the current treatment. Here, we have reported a case series of refractory FM patients successfully treated with subcutaneous tocilizumab. Their disease severity significantly improved 12 weeks later. Immuno-modulation is, therefore, a potential treatment strategy for some FM patients.

Prior meta-analysis of FM treatment demonstrated only a modest efficacy using either non-pharmacological or pharmacological therapies [5]. Moreover, low acceptability (high dropout rate) in trial participants was reported [14]. In recent decades, the immune system has been implicated in pain response. Glycoprotein 130 (gp130), a subunit of the IL-6 receptor, has been found to be expressed in the dorsal root ganglion neurons [15]. Knockdown of either IL-6 or qp130 attenuates hyperalgesia after stimuli in mice [16,17]. In rats, directly neutralizing IL-6 by injection of soluble gp130 in their spinal cords alleviate hyperalgesia that accompanies joint inflammation [18]. In addition, prior studies on FM patients found elevated circulating levels of IL-6 [1]. In line with these findings, our study on FM patients showed that subcutaneous tocilizumab had improved scores of both FIQR and fibromyalgianess at 12 weeks after treatment. In addition, their energy and sleep quality improved significantly. Nevertheless, there was only a trend toward improved pain levels at 4 weeks, despite over half of them achieving at least 30% pain reduction. Tocilizumab has been approved to treat cytokine storms in COVID-19 patients [10]. It could be interesting to investigate how FM symptoms change in patients treated with tocilizumab due to COVID-19.

Neutrophils have been implicated in the generation of pain. A prior study demonstrated that neutrophil recruitment accounted for carrageenan-induced mechanical pain hypersensitivity [6]. Another study also showed that inhibition of elastase in recruited neutrophils abrogated joint pain in an experimental model of monoiodoacetate-induced osteoarthritis [7]. Our preliminary work in neutrophils of FM patients suggested that *C3AR1* had been upregulated by as much as 4-fold. Complement 3a (C3a) is a bioactive product of C3 cleavage, and it promotes through the C3a receptor the inflammatory responses in neutrophils [19]. In addition, the C3a-C3a receptor can promote neutrophil recruitment depending on the tissue context. Previous studies on tocilizumab demonstrated that it had decreased neutrophil survival, energy availability, oxidative burst, and phagocytosis [9]. We also observed the possible down-regulation of *secreted phosphoprotein 1* (*SPP1*) in neutrophils after tocilizumab treatment. Secreted phosphoprotein 1 produces a strong chemotactic effect on infiltrating immune cells, including neutrophils [20,21]. Nonetheless, our observations need validation in an independent cohort.

Our study has several limitations. First, the sample size was small, and our findings need confirmation with studies of a larger sample. Second, this is an observational study subject to possible placebo effects. Third, the follow-up periods of FM patients were relatively short. Fourth, the improvement in symptoms, though statistically significant, may not be clinically significant, and some patients had a flare of their symptoms at 12 weeks. The therapeutic efficacy of tocilizumab in these patients is therefore doubtful. Nevertheless, our study still sheds light on the newer treatment options for these refractory FM patients. Last, our FM patients were all Han Chinese, and the extrapolation of our results to other ethnic groups should be performed cautiously.

In conclusion, our results unveiled the potential efficacy of subcutaneous tocilizumab on some refractory FM patients. Further randomized trials are required.

## Figures and Tables

**Table 1:** Baseline characteristics of fibromyalgia patients receiving subcutaneous tocilizumab.

Patient	1	2	3	4	5	6
Age (years/gender)	52/female	52/female	43/female	58/female	36/male	49/female
Concomitant rheumatic diseases	No	No	Rheumatoid arthritis	Hypereosinophilic syndrome	Ankylosing spondylitis	Sjogren’s syndrome
Number of painful tender points ^a^	18	11	18	17	15	18
Disease duration (years)	3	3	4	6	2	7
Fibromyalgianess scale	26	27	24	30	30	28
Revised Fibromyalgia Impact Questionnaire score	78.5	81.5	92.2	90	71.3	72.5
ESR (mm/h)/CRP (mg/dl)	31/0.079	3/0.04	12/0.062	14/0.055	3/0.11	9/0.592
Level of pain	8	10	10	10	7	10
Depression	-	-	+	+	+	+
Synovitis by 40-joint sonography	No	No	No	Bilateral elbows grade 1 synovitis	No	No
Medications for fibromyalgia	Pregabalin 450 mg/day	Pregabalin 450 mg/day and tramadol 37.5 mg/acetaminophen 325 mg 8 tablets/day	Pregabalin 450 mg/day	Pregabalin 75 mg/day, 37.5 mg/acetaminophen 325 mg 6 tablets/day	Pregabalin 450 mg/day, duloxetine 60 mg/day, and 37.5 mg/acetaminophen 325 mg 8 tablets/day	Pregabalin 75 mg/day and duloxetine 60 mg/day
Dose of tocilizumab	126 mg every 2 weeks	126 mg every 2 weeks	126 mg every 2 weeks	126 mg every 4 weeks	126 mg every 2 weeks	126 mg every 4 weeks

^a^ based on the American College of Rheumatology 1990 classification criteria for fibromyalgia.

**Table 2:** Comparison of outcomes between baseline, 4, and 12 weeks after subcutaneous tocilizumab therapy in fibromyalgia patients.

Outcomes, Median (IQR)	Baseline	4 Weeks	12 Weeks
WPI	18 (14.5, 19.0)	11.5 (7.5, 15.3) *	12 (9.0, 13.8) *
SSS	11 (9.0, 11.3)	8 (7.0, 10.0) *	9 (7.3, 10.5)
Fibromyalgianess score	27.5 (25.5, 30.0)	19 (15.8, 23.8) *	21.5 (16.3, 23.5) *
FIQR	80 (72.2, 90.5)	55.8 (51.8, 69.8) *	67.5 (57.5, 74.0) *
Pain	10 (7.8, 10.0)	4.5 (3.8, 7.3)	8 (7.8, 9.0)
Energy	9 (8.3, 10.0)	6 (3.8, 7.3)	7.5 (5.0, 8.5) *
Sleep quality	10 (9.8, 10.0)	8 (4.8, 9.3) *	8 (5.8, 8.5) *

FIQR, the revised Fibromyalgia Impact Questionnaire score; IQR, interquartile range; SSS, symptom severity score; WPI, widespread pain index. * *p* < 0.05.

## Data Availability

Study data are available upon request of the corresponding author.

## Supplementary Materials

The following supporting information can be downloaded at https://www.mdpi.com/article/10.3390/biomedicines11071774/s1. Figure S1. Venn diagram of numbers of differentially expressed genes in neutrophils of patients 1 and 2 before and after tocilizumab treatment by pairwise comparisons; Table S1: Forty-joint sonography of fibromyalgia patients receiving subcutaneous tocilizumab; Table S2. Differentially expressed genes in neutrophils from primary fibromyalgia patients when compared with matched healthy controls; Table S3. Overlapping differentially expressed genes in neutrophils from primary fibromyalgia patients before and after tocilizumab treatment.
