# Supplementary material for: Subcutaneous Tocilizumab May Be Effective in Refractory Fibromyalgia Patients
Source: Biomedicines. 2023 Jun 21;11(7):1774. doi: 10.3390/biomedicines11071774 (PMC10376766; doi:10.3390/biomedicines11071774)

**Table S1.** Forty-joint sonography of fibromyalgia patients receiving subcutaneous tocilizumab.

| Patient number | Baseline | | | 12 weeks after tocilizumab therapy | | |
|---|---|---|---|---|---|---|
| | Gray scale score | Power Doppler score | Synovitis score | Gray scale score | Power Doppler score | Synovitis score |
| 1 | 4 | 0 | 4 | 11 | 0 | 11 |
| 2 | 18 | 0 | 18 | 16 | 0 | 16 |
| 3 | 13 | 0 | 13 | 12 | 0 | 12 |
| 4 | 3 | 2 | 3 | 4 | 2 | 4 |
| 5 | 4 | 0 | 4 | 4 | 0 | 4 |
| 6 | 15 | 0 | 15 | 15 | 4 | 16 |

**Table S2.** Differentially expressed genes in neutrophils from primary fibromyalgia patients when compared with matched healthy controls.

| **Gene** | **Fold change (Fibromyalgia/Control)** | ***P* Value** | **False discovery rate** |
|---|---|---|---|
| *TSPAN13* | 0.179522125 | 2.15E-12 | 6E-08 |
| *C3AR1* | 3.830945028 | 7.41E-09 | 0.000103 |
| *PI3* | 1.980829949 | 1.02E-07 | 0.000953 |

**Table S3.** Overlapping differentially expressed genes in neutrophils from primary fibromyalgia patients before and after tocilizumab treatment.

| | Patient 1 | | | Patient 2 | | |
|---|---|---|---|---|---|---|
| **Gene** | **Fold change** | **P value** | **False discovery rate** | **Fold change** | **P value** | **False discovery rate** |
| LGALS2 | 7.028383767 | 0.003394436 | 0.014616555 | 186.5038397 | 7.447E-07 | 0.000128 |
| LY86 | 88.42916002 | 0.001268294 | 0.006574814 | 13.65099679 | 0.0007946 | 0.033025 |
| CD19 | 5.534619948 | 0.000672116 | 0.003929602 | 12.01058567 | 1.743E-05 | 0.001759 |
| MS4A7 | 2.937024377 | 0.005100126 | 0.020145058 | 11.74482265 | 3.369E-07 | 6.08E-05 |
| HLA-DQA2 | 5.55926675 | 9.45135E-06 | 0.00011219 | 11.53253312 | 2.951E-05 | 0.002664 |
| NAPSB | 3.229500209 | 0.00179023 | 0.00878318 | 10.37625069 | 9.198E-08 | 2.21E-05 |
| SLC38A7 | 21.18231508 | 4.812E-06 | 6.31868E-05 | 8.818694785 | 2.847E-05 | 0.002635 |
| CTSL | 5.534619948 | 0.000672116 | 0.003929602 | 8.664217877 | 0.000701 | 0.030535 |
| IRF8 | 2.057811348 | 0.014866261 | 0.047401704 | 6.937961735 | 2.825E-09 | 1.08E-06 |
| LOXL1 | 4.32228728 | 4.42692E-08 | 1.0676E-06 | 6.681354442 | 0.0001036 | 0.007366 |
| NR4A1 | 2.78402977 | 7.01099E-07 | 1.20406E-05 | 6.490927845 | 4.847E-06 | 0.000633 |
| SH3TC1 | 3.80297192 | 6.84948E-06 | 8.49806E-05 | 6.144091736 | 6.969E-07 | 0.000121 |
| CD79A | 2.779812595 | 0.000187747 | 0.001372543 | 5.733523958 | 2.489E-07 | 4.68E-05 |
| CD22 | 2.500925728 | 0.000847044 | 0.004740266 | 5.674941111 | 1.054E-07 | 2.41E-05 |
| TCL1A | 4.647143286 | 0.001177692 | 0.006198807 | 3.892775041 | 0.0008778 | 0.035427 |
| CPVL | 3.836499308 | 5.41541E-05 | 0.000483162 | 3.800321003 | 0.0006087 | 0.02773 |
| INF2 | 2.44716507 | 0.000269375 | 0.001852816 | 2.843950711 | 0.0013391 | 0.048356 |
| IGKC | 3.115647819 | 2.51314E-07 | 4.89848E-06 | 2.479709094 | 2.145E-07 | 4.27E-05 |
| IGHG1 | 3.475272749 | 0.001279284 | 0.00662106 | 2.198487339 | 0.0008683 | 0.035253 |

| | | | | | | |
|---|---|---|---|---|---|---|
| FCRL1 | 2.745142635 | 3.59433E-05 | 0.000344221 | 2.171076012 | 0.0009875 | 0.038496 |
| GGT5 | 2.050867393 | 0.012050518 | 0.040242688 | 2.090456466 | 0.0003108 | 0.016856 |
| S100A10 | 2.832958633 | 1.4312E-06 | 2.23685E-05 | 2.014238077 | 0.0008519 | 0.034687 |
| SPP1 | 0.441432763 | 0.002500939 | 0.011391689 | 0.461834234 | 8.811E-05 | 0.006431 |

**Figure S1.** Venn diagram of numbers of differentially expressed genes in neutrophils of patient 1 and 2 before and after tocilizumab treatment by pairwise comparisons.

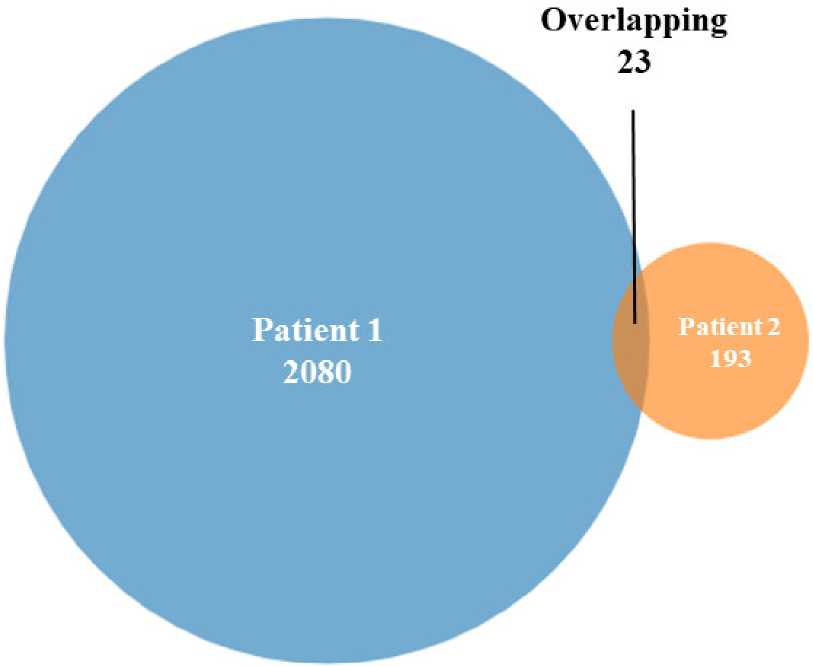

Supplement: Supplementary file 1 [file biomedicines-11-01774-s001.zip › biomedicines-2458049-supplementary.pdf]
